# Supplementary material for: Resolving the full spectrum of human genome variation using Linked-Reads
Source: Genome Res. 2019 Apr;29(4):635–45. doi: 10.1101/gr.234443.118 (PMC6442396; doi:10.1101/gr.234443.118)
Supplement: Supplemental Material [file supp_gr.234443.118_Supplemental_longranger-2.2.zip › longranger-2.2.tar.bz2!/lr2.2-public/lib/stan/cmdstan/stan/doc/api/html/messages_8hpp_source.html]

Stan: src/stan/common/recorder/messages.hpp Source File


|  |  |
| --- | --- |
|  | Stan  2.5.0  probability, sampling & optimization |


- Main Page
- Related Pages
- Namespaces
- Classes
- Files

- File List
- File Members

All Classes Namespaces Files Functions Variables Typedefs Enumerations Enumerator Friends Macros Pages

- src
- stan
- common
- recorder

messages.hpp

Go to the documentation of this file.

1 #ifndef STAN\_\_COMMON\_\_RECORDER\_\_MESSAGES\_HPP

2 #define STAN\_\_COMMON\_\_RECORDER\_\_MESSAGES\_HPP

3

4 #include <ostream>

5 #include <string>

6 #include <vector>

7

8 namespace stan {

9  namespace common {

10  namespace recorder {

11  class messages {

12  private:

13  std::ostream \*o\_;

14  const bool has\_stream\_;

15  const std::string prefix\_;

16

17  public:

18  messages(std::ostream \*o, std::string prefix)

19  : o\_(o), has\_stream\_(o != 0), prefix\_(prefix) { }

20

21  messages(std::ostream \*o)

22  : o\_(o), has\_stream\_(o != 0), prefix\_("") { }

23

24  template <class T>

25  void operator()(const std::vector<T>& x) {

26  // no op

27  }

28

29  void operator()(const std::string x) {

30  if (!has\_stream\_)

31  return;

32  \*o\_ << prefix\_ << x << std::endl;

33  }

34

35  void operator()() {

36  if (!has\_stream\_)

37  return;

38  \*o\_ << std::endl;

39  }

40

41  bool is\_recording() const {

42  return has\_stream\_;

43  }

44

45  };

46  }

47  }

48 }

49

50 #endif

stan::common::recorder::messages::operator()

void operator()(const std::string x)

**Definition:** messages.hpp:29

stan::common::recorder::messages::messages

messages(std::ostream \*o)

**Definition:** messages.hpp:21

stan::common::recorder::messages::operator()

void operator()()

**Definition:** messages.hpp:35

stan::common::recorder::messages::is\_recording

bool is\_recording() const

**Definition:** messages.hpp:41

stan::common::recorder::messages::operator()

void operator()(const std::vector< T > &x)

**Definition:** messages.hpp:25

stan::common::recorder::messages

**Definition:** messages.hpp:11

stan::common::recorder::messages::messages

messages(std::ostream \*o, std::string prefix)

**Definition:** messages.hpp:18


---

[ Stan Home Page ]

*© 2011–2014,
Stan Development Team.*
